# Mobile Applications in Breast Cancer Postoperative Care: A Scoping Review

**DOI:** 10.1002/cam4.70444

**Published:** 2024-12-16

**Authors:** Maryam Alidadi, Reza Rabiei, Atieh Akbari, Hassan Emami, Seyed Mohsen Laal Mousavi

**Affiliations:** ^1^ Department of Health Information Technology and Management, School of Allied Medical Sciences Shahid Beheshti University of Medical Sciences Tehran Iran; ^2^ Obstetrics and Gynecology, Cancer Research Center Shahid Beheshti University of Medical Sciences Tehran Iran; ^3^ Department of Health Information Management, School of Allied Medical Sciences Tehran University of Medical Sciences Tehran Iran

**Keywords:** breast neoplasms, mhealth, mobile application, postoperative care

## Abstract

**Background:**

The utilization of mobile application in postoperative care for breast cancer patients has seen a significant rise in recent years. This study aimed to synthesize the literature to identify the features of breast cancer postoperative care mobile applications.

**Methods:**

This scoping review was conducted using the framework developed by Arksey and O'Malley. All articles published from inception until July 25, 2024, were searched in the PubMed, Scopus, Web of Science, IEEE, and Cochrane databases. The quality of publications was evaluated using the mixed‐methods appraisal tool (MMAT).

**Results:**

A total of 999 publications were found, of which 28 studies were considered in this review. Out of these studies, 14 used native apps, 14 used hybrid apps. Nine features were used in applications, and Tracker, Tailored Education, and Community Forum were the most repetitive features. In five studies, various devices and sensors, like Bluetooth and GPS, were utilized in mobile applications to monitor physical activity, stress levels, heart rate, sleep patterns, and calorie intake.

**Conclusions:**

Mobile applications for postoperative breast cancer care encompass a range of features. In a co‐design approach, understanding patients' required features could help to develop usable applications to improve the postoperative care for breast cancer patients.

## 1 Introduction

Breast cancer is the most widespread and common malignant tumor globally, posing a significant threat to the health of the world's population, particularly women, as it is a leading cause of morbidity, mortality, and associated disability [1, 2]. In its 2022 report, the World Health Organization (WHO) indicated that approximately 2.3 million women were diagnosed with breast cancer, resulting in 670,000 fatalities worldwide [3]. Projections indicate that by 2070, there may be approximately 4.4 million new cases [4, 5]. In 2024, it is estimated that there will be 310,720 new cases of invasive breast cancer diagnosed in women in the United States, resulting in approximately 42,250 deaths due to the disease [6]. Surgical interventions constitute a fundamental component of primary treatment for breast cancer. Both breast‐conserving surgery (BCS) and mastectomy are recognized as effective local management strategies for early‐stage invasive breast cancer [7].

Various therapeutic strategies are presently accessible for the management of breast cancer. Surgical intervention stands as a prevalent treatment modality for the majority of women [8]. Hence, surgical intervention choices, physiological alterations resulting from surgical procedures, and health‐related considerations are pertinent to most patients. Surgical procedures have the potential to give rise to enduring complications, the nature of which is contingent on the type of surgery and adjuvant therapy [9]. Women undergoing breast cancer surgery may face complications from medical procedures [10]. Such complications can impact both physical and mental health, leading to alterations to lifestyle and the quality of life [11]. Breast cancer patients require diligent care to mitigate these adverse complications, enhance their quality of life, and limit hospital readmission [12]. Cancer patients and survivors have many responsibilities related to managing their recovery and illnesses; therefore, healthcare professionals must equip them with the knowledge and skills necessary to manage their disease [13]. While traditional in‐person interventions can benefit cancer care [14, 15]. Mobile applications has demonstrated significance in managing patients with cancer, especially in follow‐up and supportive care [16, 17] and effective in mitigating stress and enhancing overall well‐being among young adult cancer survivors [18].

Mobile applications have seen significant growth within the healthcare sector, coinciding with the increasing prevalence of smartphone usage in the community. In the upcoming decade, mobile learning through personal electronic devices is anticipated to emerge as the most impactful technology for enhancing cancer patient education [19].

Research indicates that mobile applications that involve virtual communities, symptom monitoring, and disease‐specific information effectively engage adolescents and young adults (AYAs) with breast cancer, addressing their unique needs and preferences [20, 21, 22]. Symptom and pain tracking features in applications have received favorable assessments from AYAs diagnosed with breast cancer, who reported that these functionalities operated effectively and enhanced the quality of supportive cancer care [17, 21, 23, 24]. A vast number of studies have been conducted on mobile application for breast cancer patients due to the significant role this technology plays in delivering healthcare services [25, 26, 27]. The increasing attention to this area of research can be linked to the ease of use and user‐friendly aspects of smartphones, along with the beneficial results seen from using mobile technology for breast cancer patients [27, 28, 29].

By using mobile applications, patients can promptly report treatment‐related complications. Furthermore, these applications offer readily accessible healthcare information at minimal expense, motivating patients to achieve health professional‐recommended goals with the added benefit of immediate feedback [30, 31]. As the number of breast cancer surgeries has increased, there is a need to extend high‐quality patient‐centered care beyond hospitals [32]. Therefore, digital health solutions are leading the way, with the goal of enhancing connectivity between patients and healthcare providers and promoting information sharing and communication [33, 34]. The adoption of mobile applications as an effective means of enhancing adherence to medical treatment is steadily increasing [35]. Furthermore, these applications serve as valuable tools for providing a wide variety of educational and behavioral interventions, allowing health care providers to track patient [36].

Thus, the main objective of this review is to identify studies on the use of mobile applications by breast cancer patients during postoperative care. Moreover, the secondary objectives are to categorize and identify the features of mobile applications.

Our findings provide valuable insights to mobile application developers, healthcare professionals, and stakeholders concerning the features of applications in breast cancer postoperative care. This study will assist in developing practical applications to support patients in their care management.

## 2 Methods

### 2.1 Study Design

Scoping reviews are intended to identify, gather, and summarize relevant data from research studies that pertain to a particular subject, in order to uncover essential ideas and illustrate the existing literature on that topic. To enhance comprehension of the scope and coverage of evidence regarding the features of mobile applications, this review was conducted based on the framework suggested by Arksey and O'Malley, as specified in the Joanna Briggs Institute (JBI) guidance [37].

This framework offers a systematic approach to conducting a scoping review, ensuring that the review process is transparent and comprehensive [37, 38]. The following five steps are suggested according to this framework: the first four steps are mandatory to guarantee the reliability and dependability of the review, while the final step is considered optional.

The authors adhered to the Preferred Reporting Items for Systematic Review and Meta‐Analysis Extension for Scoping Reviews (PRISMA‐ScR) as a framework for this scoping review [39].

#### 2.1.1 Identifying the Research Question

A scoping review uses questions to guide the initial identification and selection of relevant studies. These questions should include the population, context, and concept components as per the guidelines. Thus, the primary research question is: What mobile applications have been developed for postoperative care in breast cancer and what are the special features of these mobile applications?

#### 2.1.2 Identification of Relevant Studies

Searches were performed in several databases, including the Web of Science, PubMed, IEEE, Scopus, and Cochrane databases. We used the MeSH terms and multiple keywords. The search strategy employed the following keywords.

((“mhealth”[Title/Abstract] OR “Mobile applications”[Mesh] OR “Smartphone”[Mesh] OR “App”[Title/Abstract]) AND (“Breast Tumor”[Title/Abstract] OR “Breast Neoplasm”[Mesh] OR “Breast Carcinoma”[Title/Abstract]) AND (“Postoperative”[Title/Abstract] OR “Postoperative Care “[Mesh] OR “surgery”[Title/Abstract]))

The search included articles published from inception up to July 25, 2024. EndNote X9 was used to handle the screening processes of the articles.

#### 2.1.3 Study Selection

The initial step involved screening the retrieved articles based on their titles and abstracts. Two independent authors assessed all titles and abstracts, and any disagreements between the two authors were resolved by consulting the senior author. Once the senior author approved the remaining articles, two independent reviewers carried out a full‐text evaluation based on the study's inclusion and exclusion criteria (Table 1). In order to comprehensively identify and analyze the features of mobile applications, protocol studies were also included in this review. The process of selecting studies was facilitated using the Rayyan Web platform. This platform is an online tool created primarily to support blind screening procedures for reviews [40].

**TABLE 1:** Eligibility criteria.

**Inclusion criteria** Original studies focusing on designing and developing of mobile health applications. Published in English from inception up to July 25, 2024. Enrolled patients undergoing surgery for breast cancer and receiving postoperative care. Highlighting the features of mobile applications. **Exclusion criteria** Case studies, reviews, conference abstracts, and guidelines. Participants received breast cancer treatments other than surgery (i.e., chemotherapy, radiotherapy, and targeted therapy). Interventions focused on breast cancer prevention and screening. Studies on telecommunication technology (such as games, websites, phone alerts, and computer‐based online programs).

#### 2.1.4 Charting Data

The data derived from the articles, encompassing the study design, participant age range (or mean), length of intervention, application features, outcome or results, data collection tool, connectivity, target group, name of application, operating system, type of application, purpose of study, criteria used in application evaluation, and general article details (article title, DOI, authors, country, and year), were meticulously recorded in Excel Version 2304. The findings were visually structured and presented in a narrative format.

Moreover, the key features of mobile applications were analyzed and categorized as per Mendiola et al. [41] into the subsequent categories: general education, gamification, export of data, tailored education, reminder, plan or orders, community forum, tracker, social media, addressed symptoms, usability, and cost.

### 2.2 Quality Assessment

To evaluate the methodological quality of the included articles, the Mixed Methods Assessment Tool (MMAT) version 2018 [42] was used.

MMAT is a valuable and efficient approach for evaluating the quality of systematic reviews of mixed studies. The quality of five study types, including non‐randomized controlled trials, randomized controlled trials, quantitative descriptive, qualitative research, and mixed method studies, is evaluated using MMAT. There are five questions for each form of study and two screening questions for all categories. The quality assessment of the included studies was rated as “yes,” “no,” or “can't tell,” based on how well the study followed the set criteria. Tool developers believe that the ratings for each category should not be used to determine the overall score.

Furthermore, they advise against dismissing studies because of their quality. Using the MMAT checklist, three authors (MA, RR, and HE) assessed the quality of the included articles. Quality evaluation of the included studies was not carried out to remove these studies from the research to meet the objectives of this investigation. However, quality assessment results were used during data analysis to determine the limitations of the included studies.

## 3 Results

### 3.1 Study Selection

**FIGURE 1:**
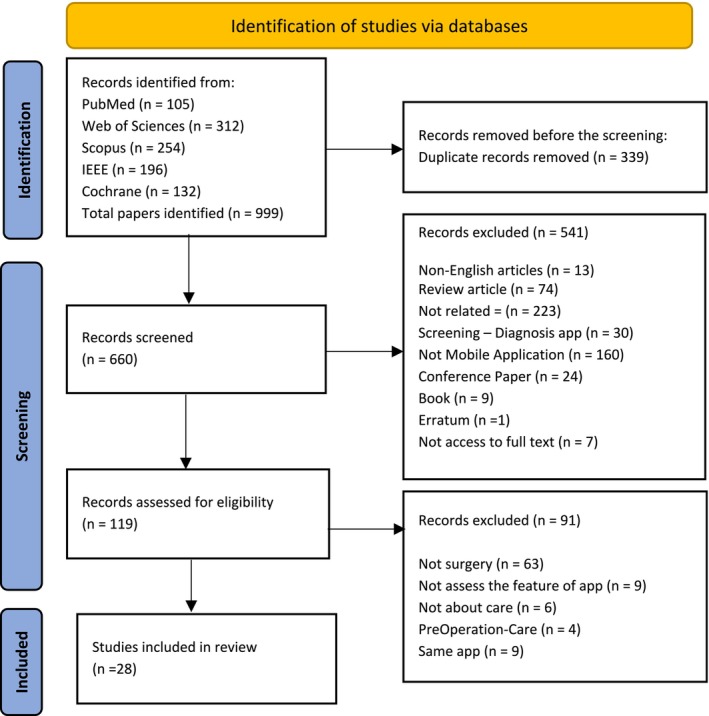
Flow diagram of the identification, screening, and inclusion of studies for review.

During the search process in electronic databases, 999 articles were retrieved. After eliminating duplicates, the 660 remaining articles were assessed using their titles and abstracts. Out of these, 541 articles were excluded for not meeting the predefined inclusion criteria. Consequently, 119 articles underwent full‐text screening, and a further review using predefined exclusion criteria led to the exclusion of studies unrelated to surgery, web‐based software, lack of assessment of application features, or care related to preoperative and same applications. This removed 91 articles from the 119 studies. Eventually, a total of 28 studies were included in the final review. The process is illustrated in the flow diagram in Figure 1.

### 3.2 Study Characteristics

The articles included in the review encompassed a publishing timeframe from January 2016 to July 2024. All articles were reviewed, covering breast cancer patients and survivors. Only one study focused on prostate cancer and breast cancer [43].

**FIGURE 2:**
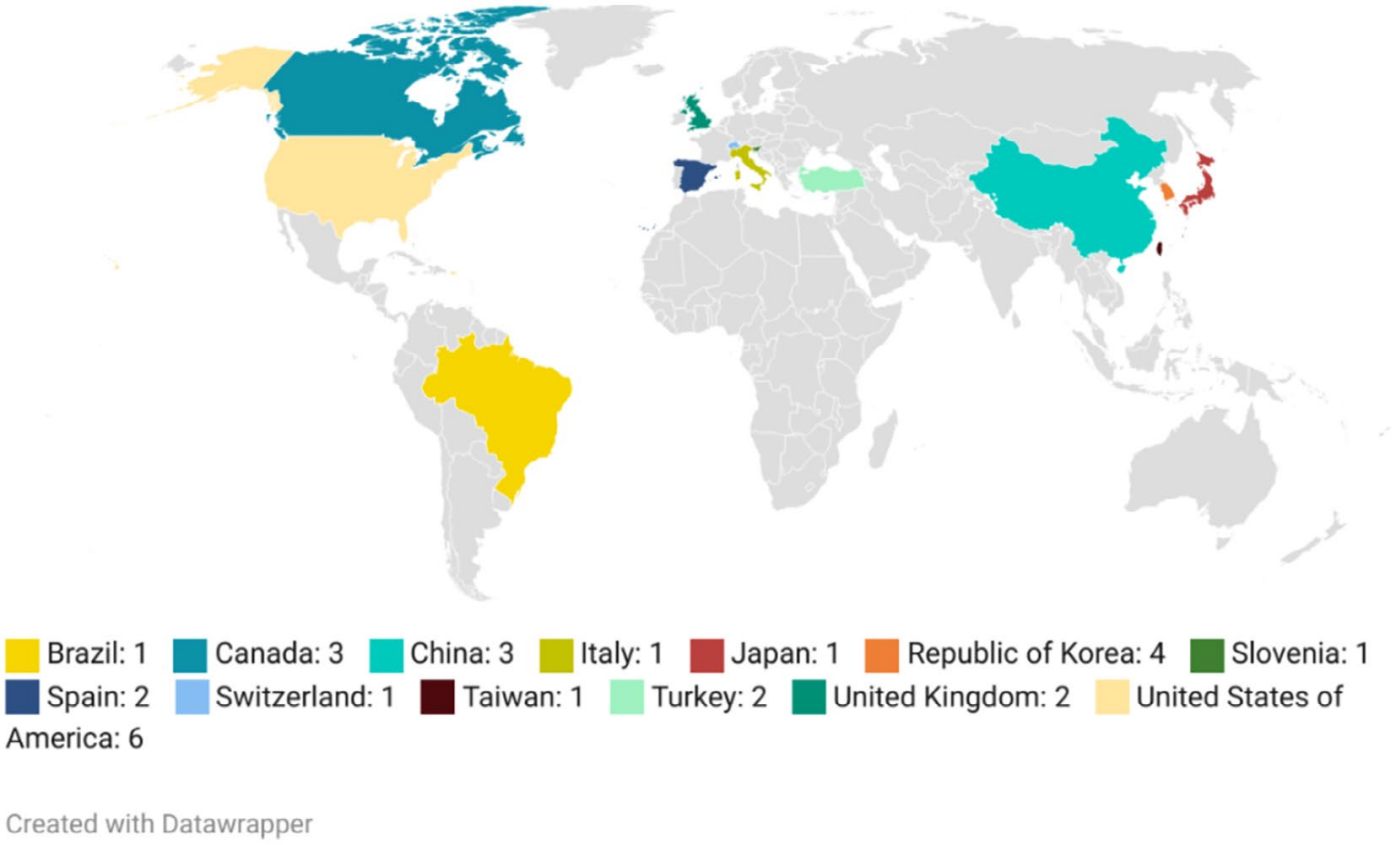
Distribution of published studies.

As shown in Figure 1, the included studies were conducted in Asia (9/28, 32.14%), North America (9/28, 32.14%), Europe (9/28, 32.14%), and South America (1/28, 3.57%) (Figure 2). The sample size in the studies varied between 15 and 4475, 42.85% (12/28) had fewer than 50 participants, 25% (7/28) had 50 to 100 participants, and 28.57% (8/28) had over 100 participants. By contrast, in only one study, the number of participants was not specified.

The age range of individuals was 18–90 years old. In categorizing the breast cancer studies based on their inclusion of the AYA population, defined by the NCI as those aged 15–39, we observe two distinct groups. Articles that include the AYA group are those with age ranges starting below 39 or encompassing this range [31, 32, 44, 45, 46, 47, 48, 49, 50, 51, 52, 53, 54]. On the other hand, articles that do not include the AYA group are those with age ranges exclusively above 39 [55, 56, 57, 58, 59]. Several articles [43, 60, 61, 62, 63, 64, 65, 66, 67, 68] did not specify age ranges and thus could not be definitively categorized.

Most studies focused on breast cancer patients undergoing surgery; seven studies (25%) examined surgery, radiation, and chemotherapy in these patients [31, 45, 48, 49, 58, 64, 65]. Concerning the length of intervention, one study managed for 1 day [56], one for 2 weeks [50], one for 3 weeks [65], one for 6 weeks [49], three for 1 month [52, 62, 68], five for 2 months [44, 48, 55, 64, 66], five for 3 months [31, 45, 53, 63, 67], one for 18 weeks [58], five for 12 months [32, 43, 46, 47, 57, 58], and 44 months [59] (Table 2).

**TABLE 2:** Description of the characteristics of the studies reviewed.

Author/year	Country	Aim of the study	Participant number	Age (years)	Target group	Study design	Data collection tool	Length of intervention
Harder et al. /2017 [55]	UK	Development of a mobile application to enhance the management of upper limb dysfunction (ULD) in individuals receiving breast cancer treatment	*N* = 15 Phase 1 (*N* = 9) Phase 2 (*N* = 4)	Age: 47–65 Mean = 52.3	Post‐surgery patient	Development	Questionnaire focus groups	2 months
Zhang et al./2018 [43]	UK	Development and evaluation of an application with a distinctive user interface that addresses the limitations of existing, disjointed resources by enabling access, gathering, exchange, and analysis of data	*N* = 187	Not mention	Post‐surgery patient (prostate and breast cancer)	Pre–post comparison	Workshops remote testing interview by phone questionnaires	12 months
Imai et al./2019 [44]	Japan	Develop and evaluate a mobile application for problem‐solving therapy (PST) among female breast cancer survivors	*N* = 37	Age: 20–49 Mean = 44 ± 5	Post‐surgery patient	Feasibility	Japanese version of the Concern About Recurrence Scale (CARS‐J) Hospital Anxiety and Depression Scale (HADS) EuroQoL‐5 (EQ‐5D) functional assessment of chronic illness therapy–Spiritual Wellbeing‐12 (FACIT‐Sp‐12) supportive care needs survey–short form 34 (SCNS‐SF34) social problem‐solving inventory revised–short form (SPSI‐R: SF) interviews	2 months
Hou et al./2020 [45]	Taiwan	Examine the quality of life (QoL) following use of the BCSMS app among women diagnosed with breast cancer	*N* = 112Intervention group (*N* = 53) control group (*N* = 59)	Age: 20–64	Post‐surgery Chemotherapy Radiotherapy Hormone therapy patients	Randomize controlled trial	The Taiwanese Chinese iteration of two Quality‐of‐Life Questionnaires (QLQs) initially formulated by the European Organization for Research and Treatment of Cancer (EORTC): the EORTC BC‐Specific QLQ (QLQ‐BR23), and the EORTC QLQ Core 30 (QLQ‐C30), version 3.	3 months
Lim et al./2021 [60]	Republic of Korea	Development of a personalized mobile application for women with breast cancer to facilitate self‐management	Not mention	Not mention	Post‐surgery patient	Development	Meeting and discussion in phase 2	Not mention
Ormel et al./2021 [56]	Canada	Designing and receiving initial feedback on a mobile application that facilitates access to information about surgery for breast cancer patients	*N* = 56 Phase 1 (*N* = 47) Phase 2 (*N* = 4) Phase 4 (*N* = 5)	Age: 51–66	Post‐surgery patient	Development and usability	E Health Impact Questionnaire evaluation tool (in phase4) focus group and qualitative interviews (in phase1 and 2) survey	1 day
Ponder et al./2021 [32]	North Carolina	Evaluate the feasibility of this app, a cloud‐based app for breast cancer patients and their caregivers to communicate with providers, improve patient knowledge, and track surgical outcomes	*N* = 33	Age: 37–81 Mean = 58	Post‐surgery patient	Feasibility	PROMIS‐29 survey	12 months
Baek et al./2022 [46]	Korea	Evaluate the effect of mobile apps on promoting physical activity and quality of life in patients with postoperative breast cancer	*N* = 320 Group A (*N* = 80) Group B (*N* = 80) Group C (*N* = 80) Group D (*N* = 80)	Age: 20–60	Post‐surgery patient	Protocol for a randomized clinical trial	EuroQol five‐dimension (EQ‐5D) Health‐related Quality of Life Instrument Fear of Progression Questionnaire Menopause Rating Scale questionnaire dual‐energy X‐ray absorptiometry (DXA) Patient Health Questionnaire‐9 incremental cost–utility ratio (ICUR) for measure cost‐effectiveness	6–12 months
Aydin et al./2023 [61]	Turkey	Development and assessment of a mobile app designed to aid in the self‐care of breast cancer surgery patients	*N* = 15 specialists and developers program (*N* = 5) patients (*N* = 10)	Not mention	Post‐surgery patient	Development	Not mention	Not mention
Miranda et al./ 2022 [62]	Brazil	Development and validation of a mobile app to educate patients about breast cancer surgical treatment	*N* = 32 specialist doctors (*N* = 13) patient (*N* = 19)	Not mention	Post‐surgery patient	Development and validation	Questionnaire	1 month
Hwang et al./2016 [47]	Canada	Evaluating the impact of using electronic monitoring technology and smartphone applications on hospital readmissions and unplanned visits on patient care and satisfaction	*N* = 72 intervention group (*N* = 35) control group (*N* = 37)	Age: 38–78 Mean = 60.1	Post‐surgery patient	Prospective	Online survey	12 months
Allicock et al./2021 [48]	USA	Examine the feasibility and effectiveness of a mobile app‐based, 4‐week customized physical activity and nutrition program	*N* = 22 intervention group (*N* = 13) control group (*N* = 9)	Age ≥ 18 Mean = 52 ± 9	Post‐surgery, radiotherapy, chemotherapy patient	Feasibility—randomized controlled trial	Self‐reported questionnaire a computer‐based survey	2 months
Uhm et al./2017 [31]	South Korea	Examine and compare the effects on quality of life (QOL) and physical function of a conventional program with mobile health (mHealth) and pedometer	*N* = 356 mHealth group (*N* = 179) brochure group (*N* = 177)	Age: 20–70 Mean = 50.3	Post‐surgery, radiotherapy, chemotherapy patient	Prospective, Quasi‐Randomized Trial	International Physical Activity Questionnaire‐Short Form (IPAQ‐SF): European Organization for Research and Treatment of Cancer Quality of Life Questionnaire Core 30 (EORTC QLQ‐C30) Quality of Life Questionnaire BC Module 23 (EORTC QLQ‐BR23) User satisfaction questionnaire (is used specifically in the mHealth group to assess user satisfaction)	3 months
Yanez et al./2017 [49]	USA	Examine the feasibility and effectiveness of the My Guide app in improving the quality of life related to health and reducing cancer‐related suffering	*N* = 80 intervention group (*N* = 40) control group (N = 40)	Age ≥ 21	Post‐surgery, radiotherapy, chemotherapy patient	Protocol for a randomized controlled trial	The Functional Assessment of Cancer Therapy‐Breast (FACT‐B). Functional Assessment of Cancer Therapy‐General (FACT‐G7). The Impact of Event Scale (IES) measures cancer‐specific distress. knowledge about breast cancer questionnaire communication and attitudinal self‐efficacy scale for cancer (CASEcancer) The Breast Cancer Prevention Trial symptom questionnaire (BCPT) the supportive care needs survey (SCNS) satisfaction questionnaire interview	6 weeks
Yu et al./ 2021 [57]	China	Assess the effectiveness of a smartphone application in enhancing patients' adherence to multidisciplinary treatment (MDTs) among individuals diagnosed with early‐stage breast cancer	*N* = 4475 pre‐app cohort (*N* = 2966) app non‐used cohort (*N* = 861) app used cohort (*N* = 648)	Age in three groups Pre‐app cohort: ≤ 50 App non used cohort: 50–70 App used cohort: > 70	Post‐surgery patients	Observational cohort study	The clinicopathological and demographic characteristics, such as follow‐up information, were obtained from the Shanghai Jiao Tong University breast cancer database. phone calls	12 months
Fu et al./ 2022 [63]	United States	Assess the efficacy of The‐Optimal‐Lymph‐Flow system, available on the web and mobile platforms, in addressing pain, aching, and tenderness and its impact on quality of life in breast cancer survivors	*N* = 120 intervention group (*N* = 60) control group (*N* = 60)	Not mention	Post‐surgery patients	Randomized Clinical Trial	Limb Volume Difference Measurement Using an Infrared Perometer, Pain Impact Questionnaire (PIQ‐6), The Risk Reduction Behavior Checklist, A structured self‐report checklist	3 months
Nápoles et al./ 2019 [64]	United States	Evaluate a Survivorship care planning program (SCPP) for breast cancer patients approaching the end of active treatment	*N* = 23	Mean = 55.8	Post‐surgery, radiotherapy, chemotherapy patients	Mixed methods	Emotional well‐being scale of the Functional Assessment of Cancer Therapy‐General Patient Health Questionnaire 8‐item version 6‐item Brief Symptom Inventory Somatization Scale Brief Symptom Inventory Somatization Scale 8‐item self‐efficacy for managing cancer care scale 6‐item knowledge of follow‐up care scale 5‐item subset of the Medical Outcomes Study Health Distress Scale 7‐item Patient‐Reported Outcomes Measurement Information System Cancer Fatigue Scale‐Short Form interviews	2 months
Seo et al. /2021 [50]	Korea	Provide lifestyle modification support for overweight and obese	*N* = 20	Age: All ages Mean = 51.05	Post‐surgery patients	Development	Questionnaires surveys interviews	2 weeks
Kuhar et al./2020 [65]	Slovenia	Provide patients with helpful information about symptom management during systemic therapy for early‐stage breast cancer	*N* = 91 intervention group (*N* = 46) control group (*N* = 45)	Mean = 50.9	Post‐surgery patients who were receiving chemotherapy	Non‐randomized controlled trial‐ prospective cohort study	Questionnaire European Organization for Research and Treatment of Cancer Quality of Life Questionnaire Core 30 (EOR TC QLQ C‐30) and Quality of Life Questionnaire Breast Cancer Module (EOR TC QLQ BR‐23)	4 weeks
Zhu et al. /2023 [58]	China	Develop an app for breast cancer patients undergoing radiotherapy or chemotherapy, focusing on exercise interventions and supplementing them with nutritional and psychological support	*N* = 17	Age: 42–58	Post‐surgery, radiotherapy, chemotherapy patients	Mixed methods	Post‐study System Usability Questionnaire (PSSUQ)	8–18 weeks
Dong et al./2023 [59]	China	Analyze whether breast cancer patients' adherence to treatment could be enhanced by the informational support offered by expert nurses via app	*N* = 560	Age > 50: 59.59% Age ≤ 50: 41.41%	Post‐surgery patient	Cohort—observational study	Questionnaire	44 months
Petrocchi et al. /2021 [68]	Switzerland	Development a smartphone app to help breast cancer patients navigate their diagnosis and treatment journeys	*N* = 20	Mean = 51	Post‐surgery patient	Mixed method	Interview questionnaire	1 month
Lozano‐Lozano et al./2019 [66]	Spain	Assess the feasibility of using an mHealth app to monitor changes in inflammation biomarkers and identify potential predictors of these changes in breast cancer survivors	*N* = 73	Mean = 51.35	Post‐surgery patient	Prospective quasi‐experimental pre‐post	The European Organization for Research and Treatment of Cancer Quality of Life Questionnaire Core 30 (EORTC QLQ‐C30) The user version of the Mobile Application Rating Scale (uMARS) An ad hoc clinical and sociodemographic questionnaire	2 months
Najafi et al./2024 [51]	Canada	Introducing Breamy, a prototype app that uses augmented reality (AR) to empower breast cancer patients in making informed decisions	*N* = 165	Age: 18–84	Post‐surgery patient	Development	Interview online survey	Not mention
Masiero et al. /2024 [67]	Italy	Assess the usability of the PainRELife digital health ecosystem for managing chronic pain in breast cancer patients and evaluate its effectiveness in enhancing pain self‐efficacy, shared decision‐making, and reducing pain perception	*N* = 25	Mean = 47.12	Post‐surgery patient	Pilot study	Mobile Application Rating Scale (MARS)	3 months
Saraç et al./2024 [53]	Turkey	Assess the effect of educational content delivered via mobile application on anxiety, distress, and quality of life among breast cancer patients	*N* = 82 intervention group (*N* = 42) control group (*N* = 40)	Intervention group Age: 33–66 Mean = 48.81 control group Age: 32–69 Mean = 49.30	Pre‐ and post‐surgery patient	Two‐group randomized controlled trial	Interview Patient Information Satisfaction Questionnaire Hospital anxiety and depression scale (HAD) NCCN Distress Thermometer Functional Assessment of Cancer Therapy—General (FACT‐G)	3 months
Romero‐Ayuso et al. /2023 [52]	Spain	Examines the usability of a smartphone app for occupational therapy, focusing on the needs of breast cancer survivors in daily living activities	*N* = 78	Age: 18–70	Post‐surgery patient	Cross‐sectional	System Usability Scale (SUS) Questionnaire Engagement in Meaningful Activities Survey (EMAS) Occupational Balance Questionnaire (OBQ‐E)	1 month
Tsangaris et al. / 2022 [54]	USA	Develop the imPROVE platform, consisting of a patient mobile app and clinician dashboard, to improve the adoption of PROs in breast cancer treatment	*N* = 45 patient (*n* = 28) breast cancer advisory group members (*n* = 17)	Age: All ages	Post‐surgery patient	Development	Interview Focus groups	Not mention

In five out of 28 studies (17.85%) [31, 43, 48, 60, 64], mobile applications capable of interfacing with wearable devices were utilized, along with the incorporation of a sensor in a separate study. The wearable devices involved data importation from Fitbit, Withings, and Moves accounts, connections to bluetooth‐enabled devices, and DoFit smart bands. GPS sensors were also used (Table 3).

**TABLE 3:** Description of the characteristics of mobile applications.

Author/year	Name of app	App type	Operation system	Connectivity	Evaluation type	Outcome/results
Harder et al./2017 [55]	BWell	Native	IOS	Not mention	Content functionality	The app could offer personalized information to patients and assist them in managing their clinical care, especially for those undergoing treatment recovery
Zhang et al./2018 [43]	MyHealthAvatar (MHA)	Native	Android	MHA facilitates data importation from users' Withings, Fitbit, and Moves accounts by utilizing APIs offered by these devices GPS sensors	Functionality design	MHA enables patient to view their health status, receive tailored information, personalized risk assessment, recommended fitness programs, and track their weight, fitness, calories, emotions, and sleep
Imai et al./2019 [44]	Kaiketsu‐app	Native	IOS	Not mention	Usability satisfaction feasibility	Smartphone problem‐solving therapy was positively received by cancer survivors and demonstrated a beneficial effect on their fear of cancer recurrence
Hou et al./2020 [45]	BCSMS	Native	Android and IOS	Not mention	Not mention	The app improved the quality of life for women who had received treatment for breast cancer, providing comprehensive information and supportive features for disease self‐management
Lim et al./2021 [60]	Not Mention	Hybrid	Android and IOS	The app connects to a Bluetooth‐enabled wearable device, which includes a DoFit smart band worn on the wrist	Not mention	This mobile health application can provide customized rehabilitation support throughout breast cancer care by utilizing individualized content modules and leveraging user‐specific medical data such as self‐monitoring, health information, and diet management
Ormel et al./2021 [56]	Health Experiences and Real Stories (HERS)	Hybrid	Android	Not mention	Design	Utilizing evidence‐based tools incorporating varied experiences of illness can assist women in effectively managing the overwhelming abundance of post‐diagnosis information related to breast cancer
Ponder et al./2021 [32]	Manage My Surgery (MMS)	Hybrid	Android and IOS	Not mention	Usability	By offering a platform for patients and their caregivers to engage in collaborative decision‐making with healthcare providers during treatment, this application contributes to decreased anxiety, enhanced patient safety, and improved patient satisfaction
Baek et al./2022 [46]	Three mHealth apps: Noom WalkOn Second Doctor	Native	Not mention	Not mention	Cost–utility	Utilizing mobile health applications can potentially enhance the quality of life and lifestyle for postoperative breast cancer patients
Aydin et al./2023 [61]	Not mention	Hybrid	Android	Not mention	Formative assessment	The growing popularity of web‐based programs and mobile applications in health services necessitates the active involvement of health care professionals in providing supervision and ensuring their valuable functionalities
Miranda et al./2022 [62]	OncoMasto Cirurgia	Native	Not mention	Not mention	Usability performance compatibility functionality	The mobile application designed for breast cancer patients was validated by physicians and patients, resulting in 100% agreement
Hwang et al./2016 [47]	Medeo	Hybrid	Not mention	Not mention	Usability performance compatibility functionality	The group utilizing electronic monitoring experienced notably reduced rates of hospital readmissions and unplanned visits to departments of emergency or walk‐in clinics compared to the group following conventional methods. Patients in the electronic monitoring group reported enhanced care experiences and the ability to communicate with their surgeon regarding any concerns or inquiries electronically, highlighting the potential of technology, such as smartphone applications, to enhance overall population health by relieving strain on healthcare resources
Allicock et al./2021 [48]	Creating Healthy Actions through Technology (CHAT)	Hybrid	Android	ActiGraph wGT3X‐BT accelerometer (to measure physical activity)	Usability performance compatibility functionality	On average, participants engaged with daily diaries and random sampling assessments 63 times out of 84 possible instances, with the majority finding the receipt of health information through smartphones beneficial (76%) and applicable to their daily lives (84%). The intervention resulted in modifications in eating behaviors (91%) and intentions to sustain such changes
Uhm et al./2017 [31]	Smart After Care	Hybrid	Not mention	InBodyBand pedometer	Satisfaction	Both groups exhibited enhancements in quality of life (QOL) and physical function, with the mHealth group demonstrating slightly superior QOL improvement yet no noteworthy advantages in physical function or activity
Yanez et al./2017 [49]	My Guide	Hybrid	Not mention	Not mention	Usefulness satisfaction learnability usability	My guide has the capability to enhance the quality of life and tackle the challenge of limited access to supportive care for Hispanic women undergoing recovery from breast cancer treatment
Yu et al./2021 [57]	Not mention	Hybrid	Not mention	Not mention	Not mention	The mobile application designed for the comprehensive management of patients with breast cancer has the potential to enhance patients' adherence to treatment recommendations
Fu et al./2022 [63]	The‐Optimal‐Lymph‐Flow	Hybrid	Not mention	Not mention	Not mention	By offering an affordable, technology‐driven delivery system, the proposed project aims to extend the availability of The‐Optimal Lymph‐Flow app, benefitting women with breast cancer who experience or are vulnerable to pain and symptoms associated with lymph fluid accumulation
Nápoles et al./2019 [64]	TrackC	Hybrid	IOS	Integrated activity tracker (Fitbit Zip) a wearable device	Acceptability feasibility	Preliminary results indicated improvements in fatigue levels, emotional well‐being, comprehension of post‐treatment care, and daily physical activity among participants. The user‐friendly app, which provided real‐time activity tracking and yielded instant feedback, was highly valued, with reported favorable impacts on health, such as increased exercise, weight reduction, and better digestion and sleep patterns
Seo et al./2021 [50]	Health for You	Hybrid	Android	Not mention	Usability	Based on expert and user feedback, this mobile application exhibits suitability in content, design, functionality, and quality. Its potential efficacy and clinical importance in improving the health and quality of life of overweight and obese breast cancer survivors justify further research. In conclusion, the mobile application holds promise as a valuable tool for promoting a healthy lifestyle in this specific population
Kuhar et al./2020 [65]	mPRO Mamma	Native	Android	Not mention	Exploratory analysis	This application enables patients undergoing systemic treatment for breast cancer in the early stages to better cope with symptoms and have a better quality of life
Zhu et al. /2023 [58]	Yun Dong Ru Kang	Native	Android and IOS	Not mention	Usability	This app is potentially beneficial for breast cancer patients, helping to mitigate chemotherapy's negative effects on quality of life, sleep, and depression. Before future use, the app needs enhanced exercise tools, an optimized interface, and more nutrition and diet resources
Dong et al. /2023 [59]	Not mention	Native	Not mention	Not mention	Not mention	Specialist nurses enhanced treatment adherence. Breast cancer patients prioritized treatment procedures, with 78.8% questioning the schedule, 65.9% during adjuvant therapy, and only 19.6% about follow‐up and rehabilitation
Petrocchi et al./2021 [68]	CSSI	Native	Not mention	Not mention	Feasibility Usefulness Capability to improve patient empowerment	The app garnered positive feedback from patients, especially for its role in organizing cancer management and improving doctor‐patient communication. Analysis indicated that user experience greatly enhanced patient empowerment, while interviews underscored the need for regular updates and synchronization with the hospital's schedule
Lozano‐Lozano et al./2019 [66]	Not mention	Native	Not mention	Not mention	Feasibility	The study found a significant reduction in inflammatory markers (CRP and IL‐6) after 2 months of using an mHealth app to monitor energy balance in breast cancer survivors. Changes in CRP and IL‐6 levels were associated with factors such as weight, pain, quality of life, type of tumor surgery, hormone therapy, and uMARS score
Najafi et al./2024 [51]	Breamy	Native	Android	Not mention	Usefulness	The majority of participants (90%) considered Breamy to be an effective tool for aiding decisions. Initial findings suggest that utilizing augmented reality (AR) as a decision‐support system for breast cancer patients could improve their comprehension and facilitate informed decision‐making
Masiero et al./2024 [67]	PainRELife	Native	Not mention	Not mention	Usability Effectiveness	Patients found the app easy to use, accurate, and suitable, rating it nearly 4 out of 5. The app also increased patient empowerment, with medium to high MARS subscale scores and a significant reduction in pain intensity after 3 months
Saraç et al./2024 [53]	Breast Cancer Surgery Information Guide	Native	Android	Not mention	Functionality	The intervention group using the mobile app experienced significantly lower anxiety and distress levels than the control group (*p* < 0.05), though no difference in overall quality of life was observed (*p* > 0.05). These results suggest that mobile apps can reduce anxiety and distress post‐surgery, with potential long‐term benefits for quality of life
Romero‐Ayuso et al./2023 [52]	MAIA	Hybrid	Android and IOS	Not mention	Usability	The study found that breast cancer survivors experienced challenges in daily life activities, particularly in sleep, rest, and mobility, and the MAIA app could be a valuable online tool for their rehabilitation in occupational therapy
Tsangaris et al./2022 [54]	imPROVE	Hybrid	Android and IOS	Not mention	Not mention	Feedback from stakeholder meetings, patient interviews, and focus groups led to the development of a mobile app and a clinician dashboard with patient data displays. The imPROVE app aims to transform patient care by addressing the needs of patients, clinicians, administrators, and researchers using a user‐centered design approach

It is also noteworthy that out of the 28 studies reviewed, 14 articles (50%) employed native applications and 14 articles (50%) utilized hybrid applications (Table 3). Native applications are designed for a specific operating system using platform‐specific languages and frameworks. They provide high performance, a smooth user experience, and access to device‐specific features. Web applications accessed through web browsers do not require installation on a user's device. Developed using web technologies, they are easy to maintain, offer instant updates, and have broad accessibility. Hybrid applications combine native and web elements, using web technologies and a native container for deployment on multiple platforms [69].

### 3.3 Quality Assessment

Out of all the articles, 12 met all five criteria, 11 met four, two met three, one met two, and two had indeterminate criteria due to their study design (protocol study). In every research design, the primary causes of low scores were inadequate alignment between data collection and analysis, inadequate methods to answer the research questions, and the extraction of data in qualitative studies. There was no consideration of confounding factors; the evaluators were unaware of the results and did not determine the adherence level of the participants or the significant potential for risk of bias in the quantitative studies (Appendix 1).

### 3.4 Main Features of the Mobile Applications

Mendiola et al.'s [41] classification of various features was applied to all 28 studies, as shown in Table 4. Gamification, cost, and usability were not considered in this analysis. In this study, we did not examine usability as our focus was on analyzing the core functional features of mHealth applications used in postoperative breast cancer care. The cost issue was not investigated because it is probable that most applications were given to the participants for study purposes at no cost. None of the assessed applications incorporated gamification features.

**TABLE 4:** Features for mobile applications for postoperative breast cancer patients.

Study	(1) export of data	(2) social media	(3) general education	(4) tailored education	(5) plan or orders	(6) reminder	(7) community forum	(8) addresses symptoms	(9) tracker
Harder et al. [55]			**✓**			**✓**			**✓**
Zhang et al. [43]	**✓**			**✓**	**✓**	**✓**			**✓**
Imai et al. [44]				**✓**	**✓**				
Hou et al. [45]	**✓**						**✓**	**✓**	**✓**
Lim et al. [60]				**✓**	**✓**	**✓**	**✓**		**✓**
Ormel et al. [56]				**✓**					
Ponder et al. [32]	**✓**		**✓**			**✓**			**✓**
Baek et al. [46]				**✓**			**✓**		**✓**
Aydin et al. [61]				**✓**			**✓**	**✓**	
Miranda et al. [62]			**✓**						
Hwang et al. [47]	**✓**						**✓**		**✓**
Allicock et al. [48]				**✓**					
Uhm et al. [31]				**✓**	**✓**				**✓**
Yanez et al. [49]				**✓**	**✓**			**✓**	
Yu et al. [57]					**✓**	**✓**	**✓**		
Fu et al. [63]			**✓**				**✓**	**✓**	
Nápoles et al. [64]	**✓**		**✓**			**✓**		**✓**	**✓**
Seo et al. [50]			**✓**		**✓**		**✓**		**✓**
Kuhar et al. [65]	**✓**					**✓**		**✓**	**✓**
Zhu et al. [58]		**✓**		**✓**		**✓**	**✓**		
Dong et al. [59]				**✓**	**✓**	**✓**	**✓**		
Petrocchi et al. [68]	**✓**		**✓**			**✓**			
Lozano‐Lozano et al. [66]				**✓**	**✓**				**✓**
Najafi et al. [51]			**✓**				**✓**		
Masiero et al. [67]	**✓**		**✓**					**✓**	
Saraç et al. [53]			**✓**			**✓**			
Romero‐Ayuso et al. [52]	**✓**			**✓**		**✓**	**✓**	**✓**	**✓**
Tsangaris et al. [54]			**✓**	**✓**			**✓**		**✓**
Total	9 (32.14%)	2 (3.57%)	11 (39.28%)	14 (50%)	9 (32.14%)	12 (42.85%)	13 (46.42%)	8 (28.57%)	14 (50%)

#### 3.4.1 Export of Data

This function allows users to communicate with healthcare professionals or to share data. Of the 28 studies, only nine were tailored for this function. Patient rights and control over data sharing have been investigated in a series of research studies focusing on the export of data features in mobile applications. It was discovered that patients could choose how their data was shared with relevant stakeholders, including doctors and research organizations. Additionally, patients have the option to voluntarily share their data with others, fostering a feeling of unity and encouragement [43]. Hwang et al. [47] designed an application to empower patients by enabling them to conveniently send photos of their postoperative wounds directly to their respective surgeons, facilitating prompt assessment and guidance. Another application called TrackC [64] allows users to easily share data about their cancer diagnosis and treatment, including that of clinicians, via convenient email functionalities. Petrocchi et al. [68] developed an application that allows patients to store and easily access clinical reports (e.g., radiology and blood test reports), which can be shared with other doctors if a second opinion is needed.

#### 3.4.2 General Education

This feature providing fundamental educational information on a disease or condition. This feature was reported in 11 studies. For instance, Harder et al. [55] designed an application showcasing the development of arm and shoulder exercise videos and text‐based materials to aid postoperative patients in performing exercises. Ponder et al. [32] discussed the availability of multimedia educational resources and FAQs that allow patients to access information about procedures, preparation guidelines, recovery expectations, and common concerns. Additionally, Miranda et al. [62] provide patients and their families with comprehensive information about breast cancer surgery, including issues, complications, and expected outcomes. The Health for You application also provides resources for understanding and overcoming distress in cancer survivors, including a comprehensive self‐test that covers various life areas affected by distress. It offers stress coping strategies encompassing the maintenance of a healthy lifestyle, cultivation of constructive experiences, pursuit of professional assistance, and promotion of open communication [50].

#### 3.4.3 Plan or Orders

This feature offers a roadmap to achieve the desired objective, outlining clear and practical actions to navigate the process. This particular component was found in nine different studies. According to Lim et al. [60], a mobile application recommends step intensity levels and targets for calorie burning, heart rate, and step count. For instance, it suggests reaching milestones like 5000 steps and burning 200 kcal. Throughout the application, the exercise goal and duration were determined by an algorithm based on the user's perceived exertion rate, ensuring an appropriate level of intensity. If the exercise becomes too challenging, the intensity is adjusted to maintain a suitable level of difficulty. Moreover, Yanez et al. [49] developed an application focusing on nutrition for breast cancer survivors, guiding food choices, portion control, eating out tips, and healthy recipes to promote a balanced diet. The MHA provides a range of recommended exercises that target various stages of recovery. Users have the flexibility to customize exercises according to their needs and can easily track their progress through a recorded list. Additionally, the application includes a 12‐week weight loss program that dynamically adjusts the weight loss goal based on the user's progress, continuously monitors weight changes, and provides weekly summaries and activity updates [43]. In the app developed by Dong et al. [59], patients register, begin their first treatment course, and confirm treatment‐related records after each therapy cycle. The system automatically generates a treatment schedule based on recommendations and sends periodic notifications to patients. Lozano‐Lozano et al. [66] developed an application offers specific, personalized physical exercise, and nutritional plans for breast cancer survivors.

#### 3.4.4 Reminder

A feature that encourages the user to engage in a particular action using a pre‐set alert. This feature is mentioned in 12 studies in this review. The BWell application highlights the effectiveness of a self‐setting reminder feature that aims to prompt patients daily for their next exercise session, ultimately increasing engagement and user retention [55]. Lim et al. [70] emphasized the use of alarm sounds in medication management to remind users to take their medications and record their daily smoking status. Furthermore, Ponder et al. [32] developed an application in their study that utilized push reminders to automatically notify patients of various tasks, such as appointment confirmation and completion of preoperative screening. In another study, Kuhar et al. [65] focused on the application's ability to send reminder notifications to patients, encouraging them to record their symptoms regularly.

#### 3.4.5 Community Forum

A feature that serves as a message board or chat room where people with similar interests may exchange questions and experiences, whether they are patients with comparable medical issues or caregivers. In the present study, this feature was repeated in 13 studies. Examination of relevant studies indicated that most applications enable real‐time communication between patients and specialist nurses, allowing access to consultations and guidance throughout their treatment and fostering the exchange of patient opinions and support [50, 57]. For instance, Lim et al. [60] designed an application that provides consultations on exercise and nutrition, offering users the option to seek advice from healthcare professionals through text messages, voice recordings, and images, with responses provided within a 24‐h timeframe. The “Noom” application offers consultation chat with a human coach specifically focused on daily diet, while the “Doctor” application provides online consultations with nutritionists and exercise specialists [46]. Aydin et al. [61] reveal an application that offers counseling services, providing personalized support and guidance for specific health concerns. If additional assistance is needed, there is a section to ask questions, and the first author ensures prompt response within 6 h, ensuring that patients receive timely counseling and support. Zhu et al. [58] developed an app to facilitate patient‐to‐patient connections by creating a social forum for sharing updates and images at any time. The Breamy app includes online support groups and encourages communication among patients, facilitating sharing of personal experiences [51].

#### 3.4.6 Social Media

A feature that links users to social media platforms such as Facebook allows them to share their progress with family, friends, and other people. This feature was used in only one out of 28 studies. Zhu et al. [58] developed an app that shared scientific articles from various disciplines on popular social media platforms like WeChat and Weibo. This approach aimed to enhance patients' understanding of their condition and improve perceptions of breast cancer, ultimately helping them alleviate negative psychological states related to their diagnosis.

#### 3.4.7 Addresses Symptoms

A feature that treats and helps manage a disease that manifests as pain or other observable symptoms. The application, as discussed in the study by Aydin et al. [61], offers comprehensive sessions addressing symptom management in various settings, including hospital and home care, and survival phase symptom management, including follow‐up care, treatment options, nutrition, and emotional well‐being. One study emphasized the “Managing My Symptoms” section, providing support and solutions for the post‐cancer treatment of physical and psychological symptoms [49]. The Optimal‐Lymph‐Flow application assists with chronic pain and related symptoms, monitoring, and managing aspects such as tenderness, BMI, lymphedema symptoms, and overall quality of life [63]. The Mamma mPRO application underscores the simplified approach to symptom management, offering a severity scale for each symptom, and tailored advice from healthcare professionals based on the severity level chosen by the patient [65]. Additionally, Hou et al. [45] developed an application with a warning system that triggers patients to rate their emotions as poor. These warnings indicate abnormal data, enabling prompt support and intervention. This feature of the PainRELife app is focused on the ongoing assessment and management of chronic pain symptoms. It includes validated questionnaires and tools that patients can use to evaluate their pain intensity and psychological well‐being indicators such as anxiety and depression [67].

#### 3.4.8 Tailored Education

Feature that provides education to patients based on their requirements, interests, and usage according to disease stage or disease progression. In our analysis, several studies (14 articles) incorporated this feature. In this regard, Imai et al. [44] introduced the Kaiketsu application, a psychological treatment application for cancer patients that utilizes instant messenger exchanges with cartoon characters to teach problem identification, goal setting, solution generation, solution implementation, and outcome evaluation. Additionally, another application provides updated weekly educational information on nutrition, exercise, and disease, adapting the content based on user‐entered information. The application adjusts information based on the user's type of treatment, surgery date, and chronic disease [60]. The HERS application offers personalized content through personal narratives, videos, and audio recordings to educate patients with newly diagnosed breast cancer. This application employs advanced computing engineering and machine learning to generate personalized recommendations based on the preferences and needs of women with newly diagnosed breast cancer. It provides information about other women's experiences with breast surgery through a recommendation algorithm and displays a list of relevant videos [56]. ImPROVE app offers patient‐specific education through direct links to resources tailored to the type and stage of treatment (pre‐operative, post‐operative, and managing symptoms) [54].

#### 3.4.9 Tracker

This feature enables information recording for self‐monitoring to change attitudes or behaviors to meet predefined goals. Zhang et al. [43] designed an application for self‐motivated data management and user‐centered data collection. It allows users to input both automatic and manual data from fitness sensors, such as daily weight, sleep, and questionnaire responses. The application also provides an editing panel for users to easily input or modify their data. It also offers weekly and monthly summaries to track the progress. The activity tracking application utilizes GPS sensors to monitor location, activity data, and calories burned, enabling users to review historical activity and location records. Patients in the Smart After Care application were directed to wear a pedometer continuously during their active periods, and the duration of their weekly physical activity was recorded and documented through the application [31]. Additionally, the application described in the study by Seo et al. [50] covers various dietary topics, including breast cancer, obesity, lifestyle choices, weight loss methods, and dietary control, providing a daily dietary diary for meal tracking and valuable insights from a calendar view. In another application, users can track their pain, flexibility, and mood daily using the application and visually monitor changes over time. The application also facilitates exercise tracking and progress monitoring, which are beneficial for behavioral changes [55]. The BCSMS application allows users to track medical treatments and record details, such as surgery dates, radiotherapy duration, chemotherapy medication, and physical measurements. It presents data recorded through visual graphs for ease of comprehension [45]. Nápoles et al. [64] designed an application that integrates an activity tracker, such as the Fitbit Zip, enabling users to track their progress towards personalized daily step goals. Users can access walking information, synchronize the activity tracker with the application, and view their daily step history with the added benefit of receiving visual and auditory feedback. Another application enables users to self‐assess their symptoms and track their sleep, calorie intake, stress levels, and physical activity. The monitoring screen was adjusted based on comorbidities, emphasizing chronic disease management. It also features a brief graph displaying steps, stress levels, calories, and sleep details. The application personalizes the exercise and target heart rate duration for each user based on their treatment information. During aerobic exercise, a smart band conducted real‐time heart rate monitoring. If the heart rate exceeds the designated threshold, an audible alarm will be activated, alerting them to adjust their workout intensity [60]. MAIA app allows patients to record their daily mood, pain levels, fatigue, and difficulties in performing daily tasks. This tracking enables both the patient and therapist to monitor progress over time and adjust treatment plans accordingly [52].

## 4 Discussion

This review offers a thorough overview of the application features for postoperative care for breast cancer patients from inception up to July 25, 2024. Most individuals diagnosed with breast cancer rely on mobile applications [71]. Given the widespread use of mobile applications incorporation into breast cancer treatment, it is imperative to comprehend their fundamental features. We identified 28 relevant studies showcasing a diverse range of features used across various applications. Overall, the results showed that using mobile applications in the subject of breast cancer postoperative care had a largely beneficial impact. Each application had an average of three features, ranging from one to six. The tracker, tailored education, and community forum are three of the most frequently mentioned features.

Among the studies included in this review, the tracker feature was reported most frequently in 14 of 28 studies. The tracker feature in the mobile applications in this review allows users to assess data on mood, sleep, physical activity, stress levels, pain, diet, and medical treatment. According to Cooley et al. [72], symptom tracking has been found to lead to better patient outcomes in cancer treatment. Lu et al. [73] reviewed 11 mobile applications for cancer patients, finding features for tracking symptoms, including unlisted ones, recording symptom intensity, making notes, creating summaries, and sharing data. Kapoor et al. [74] conducted an extensive assessment of mobile applications for breast cancer survivors. Of the nine applications reviewed, only four were suitable for monitoring symptoms related to breast cancer treatment. However, several of these applications lack graphical health tracking over time. Seven applications in our review investigated monitoring and tracking exercise and physical activity [31, 43, 45, 55, 60, 64, 66]. However, only two application specifically mentioned the capability to provide visual graphs [45, 54] and weekly and monthly summaries [43] for progress tracking. Not only can particular symptoms be tracked using mobile applications but daily activities and physiological indicators such as diet can also be tracked [75].

This review showed that the feature of tailored education was reported in 14 studies. A review was conducted by Richards et al. [76] to evaluate patients' utilization of mobile applications to obtain disease management information via mobile devices. The majority of education‐related aspects was treatment‐related but insufficient to fulfill patients' complete information demands for treatment and managing symptom. On the other hand, the educational elements in our review attempt to address a wider range of informational requirements, such as exercise, nutrition, disease [60], behavioral [48], and psychological ability [44]. They also provide personalized content in the form of audio recordings, videos, and personal anecdotes [56].

A 2020 study showed that providing adequate information through these applications can be beneficial because it promotes favorable outcomes, including better decision‐making, mental health, quality of life, and treatment adherence [77]. The preferences and characteristics of breast cancer patients might impact implementation and the usability of mobile applications [66]. According to Sotirova et al.'s analysis [12], adherence was much higher in treatments with individualized information and interventions with personalized information, where the users individually selected the contents. In our review, an application personalized educational content through audio, films, and personal stories for women newly diagnosed with breast cancer [56]. It uses advanced technology to make tailored recommendations based on needs and preferences.

Mobile applications offer excellent opportunities to enhance information sharing and communication between patients and physicians [78]. Researchers suggest that interactive functions must come first when developing new applications [68]. Ten mobile applications that were studied incorporated messaging feature for user engagement and healthcare support. These functionalities include consultation with healthcare professionals through text [46], voice recordings and images [60], personalized counseling services for specific health concerns [61], real‐time communication with specialist nurses [57], patient‐to‐patient connections [58], and a question‐and‐answer section [50] to facilitate patient interaction and support. Zheng et al. [79] reviewed 13 articles on cancer pain applications and found that messaging applications help patients communicate with medical professionals for real‐time intervention and improve pain relief.

Online platforms can provide a supportive environment for cancer patients, facilitating connections and offering psychological support opportunities [80]. Kapoor et al. [74] examined multiple applications and discovered that social networking, expressed via direct messaging, discussion groups, and blog posts, ranked as the second most prevalent feature. HJ et al. [81] discovered that integrating communication features into applications assists patients in sharing their experiences and offering psychological support. It encompasses online forums, anonymous blogs, and community sections for patient interaction, potentially enhancing the effectiveness of the application. In our review, one study utilized social media features that enabled patients to communicate through WeChat and Weibo [58].

The analysis of application features used by the AYA age group in breast cancer management highlights their key preferences and needs. The most frequently used feature is the tracker function, with eight mentions [31, 32, 45, 46, 47, 50, 52, 54], showing a strong preference for monitoring health metrics. Tailored education, noted nine times [31, 44, 46, 48, 49, 52, 54, 57, 59], indicates the value placed on personalized, relevant information. The plan or orders feature, appearing six times [31, 44, 49, 50, 57, 59], emphasizes the importance of structured treatment management. Community forums, also repeated eight times [45, 46, 47, 50, 51, 52, 54, 59], underscore the need for social support and peer connection. These findings underscore the importance of personalized, educational, and supportive features in breast cancer apps for the AYA demographic, suggesting that future app development should focus on these elements to improve engagement and effectiveness.

One important development in healthcare applications is the use of wearable technology and innovative devices. Accelerometers, pedometers, and multisensory systems that send data to other platforms, such as websites or mobile phones, are common tools [82]. In a systematic review by Dorri et al. [83], focusing on electronic health interventions for patients with breast cancer, 25% of the articles used these tools to assess physical activity. In our review, these devices were used to measure physical activity [31, 43, 48, 50, 60], stress level, heart rate, sleep patterns [60], and calorie intake [43].

These results emphasize the substantial potential of these applications in different areas of patient care and treatment management. The presence of various practical features in applications can positively impact patient engagement and self‐care. Overall, the results of this study indicate that a comprehensive application that addresses most aspects of patient needs can serve as a valuable tool in treating breast cancer patients.

To ensure a comprehensive examination of this domain, we did not impose limitations on the quality of the studies included in our review. Our goal was to provide a complete overview of all relevant aspects of mobile applications in this context. Although we assessed the quality of the studies, our decision to include them was not solely based on their ratings. Instead, we aimed to incorporate a diverse range of studies to achieve a thorough understanding of the features of mobile applications, thereby enhancing insights into their role in supporting breast cancer patients.

### 4.1 Limitations

Some limitations were present in this review that need to be acknowledged. First, the generalizability of our findings is limited to breast cancer patients and may not extend to other cancer populations. Future research should explore the applicability of mobile applications in the care of patients with different types of cancer. Second, this review included research protocols, which do not necessarily ensure to be completed. Lastly, some studies might not have been reviewed because this review only included research that was published in English and had full access.

## 5 Conclusions

The quantity of available mobile applications related to cancer is steadily rising. The 30 apps (in 28 studies) found in this study display notable technological advances in postoperative breast cancer care. The diversity in the features of these applications covers a wide range of care needs for this segment of society. Mobile applications are expected to revolutionize patient care by incorporating various features outlined in this review, including personalized training, patient data storage, self‐monitoring, and information sharing.

Future studies could conduct a thorough review of the efficacy of mobile applications in delivering postoperative care to breast cancer patients.

## Author Contributions

**Maryam Alidadi:** conceptualization (equal), methodology (equal), visualization (equal), writing – original draft (equal), writing – review and editing (equal). **Reza Rabiei:** conceptualization (equal), methodology (equal), project administration (equal), supervision (equal), writing – review and editing (equal). **Atieh Akbari:** methodology (equal), validation (equal), writing – review and editing (equal). **Hassan Emami:** conceptualization (equal), data curation (equal), methodology (equal), project administration (equal), supervision (equal), writing – review and editing (equal). **Seyed Mohsen Laal Mousavi:** methodology (equal), software (equal), visualization (equal), writing – original draft (equal).

## Ethics Statement

The authors have nothing to report.

## Consent

The authors have nothing to report.

## Conflicts of Interest

The authors declare no conflicts of interest.

## Supporting information


Appendix 1.

## Data Availability

The datasets used and/or analyzed during the current study available from the corresponding author on reasonable request.
