# Supplementary material for: Mobile Applications in Breast Cancer Postoperative Care: A Scoping Review
Source: Cancer Med. 2024 Dec 16;13(24):e70444. doi: 10.1002/cam4.70444 (PMC11647550; doi:10.1002/cam4.70444)
Supplement: Supplementary file 1 — Appendix 1. [file CAM4-13-e70444-s001.docx]

**Three ratings are possible under each criterion: Y = YES; N = NO; C = CAN'T TELL**

**Multimedia** **Appendix 1.** MMAT quality appraisal

|  | **Author / Year** | **Qualitative study** | | | | | **Mixed methods study** | | | | | **Quantitative non-randomized controlled trial** | | | | | **Quantitative randomized controlled trial** | | | | | **Quantitative descriptive study** | | | | |
| --- | --- | --- | --- | --- | --- | --- | --- | --- | --- | --- | --- | --- | --- | --- | --- | --- | --- | --- | --- | --- | --- | --- | --- | --- | --- | --- |
|  |  | **Item 1** | **Item 2** | **Item 3** | **Item 4** | **Item 5** | **Item 1** | **Item 2** | **Item 3** | **Item 4** | **Item 5** | **Item 1** | **Item 2** | **Item 3** | **Item 4** | **Item 5** | **Item 1** | **Item 2** | **Item 3** | **Item 4** | **Item 5** | **Item 1** | **Item 2** | **Item 3** | **Item 4** | **Item 5** |
| **1** | **Harder H / 2017** | Y | Y | Y | Y | Y |  |  |  |  |  |  |  |  |  |  |  |  |  |  |  |  |  |  |  |  |
| **2** | **Zhang X / 2018** | Y | Y | Y | Y | C |  |  |  |  |  |  |  |  |  |  |  |  |  |  |  |  |  |  |  |  |
| **3** | **Imai F/ 2019** |  |  |  |  |  |  |  |  |  |  | Y | Y | Y | N | Y |  |  |  |  |  |  |  |  |  |  |
| **4** | **Hou IC/ 2020** |  |  |  |  |  |  |  |  |  |  |  |  |  |  |  | Y | Y | Y | Y | Y |  |  |  |  |  |
| **5** | **Lim JY / 2021** | Y | Y | Y | Y | Y |  |  |  |  |  |  |  |  |  |  |  |  |  |  |  |  |  |  |  |  |
| **6** | **Ormel I / 2021** | Y | Y | Y | Y | Y |  |  |  |  |  |  |  |  |  |  |  |  |  |  |  |  |  |  |  |  |
| **7** | **Ponder M / 2021** |  |  |  |  |  |  |  |  |  |  | Y | Y | Y | C | Y |  |  |  |  |  |  |  |  |  |  |
| **8** | **Baek SY / 2022** |  |  |  |  |  |  |  |  |  |  |  |  |  |  |  | C | C | C | C | C |  |  |  |  |  |
| **9** | **Aydin A / 2023** | Y | C | C | Y | Y |  |  |  |  |  |  |  |  |  |  |  |  |  |  |  |  |  |  |  |  |
| **10** | **Miranda FD/ 2022** |  |  |  |  |  |  |  |  |  |  |  |  |  |  |  |  |  |  |  |  | Y | Y | Y | C | Y |
| **11** | **Hwang H/ 2016** |  |  |  |  |  |  |  |  |  |  | Y | Y | Y | N | Y |  |  |  |  |  |  |  |  |  |  |
| **12** | **Allicock M / 2021** |  |  |  |  |  |  |  |  |  |  |  |  |  |  |  | Y | Y | Y | C | Y |  |  |  |  |  |
| **13** | **Uhm KE / 2017** |  |  |  |  |  |  |  |  |  |  |  |  |  |  |  | Y | Y | Y | C | C |  |  |  |  |  |
| **14** | **Yanez B / 2017** |  |  |  |  |  |  |  |  |  |  |  |  |  |  |  | C | C | C | C | C |  |  |  |  |  |
| **15** | **Yu J / 2021** |  |  |  |  |  |  |  |  |  |  |  |  |  |  |  |  |  |  |  |  | Y | Y | Y | Y | Y |
| **16** | **Fu MR / 2016** |  |  |  |  |  |  |  |  |  |  |  |  |  |  |  | Y | Y | Y | Y | Y |  |  |  |  |  |
| **17** | **Nápoles AM / 2019** |  |  |  |  |  | Y | Y | Y | Y | Y |  |  |  |  |  |  |  |  |  |  |  |  |  |  |  |
| **18** | **Su-Jin Seo / 2021** | Y | Y | Y | Y | Y |  |  |  |  |  |  |  |  |  |  |  |  |  |  |  |  |  |  |  |  |
| **19** | **Kuhar CG/ 2020** |  |  |  |  |  |  |  |  |  |  | Y | Y | Y | C | Y |  |  |  |  |  |  |  |  |  |  |
| **20** | **Zhu J/ 2023** |  |  |  |  |  | Y | Y | Y | Y | Y |  |  |  |  |  |  |  |  |  |  |  |  |  |  |  |
| **21** | **Xiaojing D/ 2023** |  |  |  |  |  |  |  |  |  |  | Y | Y | Y | Y | Y |  |  |  |  |  |  |  |  |  |  |
| **22** | **Petrocchi S/ 2021** |  |  |  |  |  | Y | Y | Y | C | Y |  |  |  |  |  |  |  |  |  |  |  |  |  |  |  |
| **23** | **Lozano-Lozano M/ 2019** |  |  |  |  |  |  |  |  |  |  | Y | Y | Y | Y | Y |  |  |  |  |  |  |  |  |  |  |
| **24** | **Najafi N/ 2024** |  |  |  |  |  |  |  |  |  |  |  |  |  |  |  |  |  |  |  |  | C | N | Y | C | Y |
| **25** | **Masiero M/ 2024** |  |  |  |  |  |  |  |  |  |  | Y | Y | Y | C | Y |  |  |  |  |  |  |  |  |  |  |
| **26** | **Saraç FS/ 2024** |  |  |  |  |  |  |  |  |  |  |  |  |  |  |  | Y | Y | Y | C | Y |  |  |  |  |  |
| **27** | **Romero-Ayuso D/ 2023** |  |  |  |  |  |  |  |  |  |  |  |  |  |  |  |  |  |  |  |  | Y | Y | Y | C | Y |
| **28** | **Tsangaris E/ 2022** | Y | Y | Y | Y | Y |  |  |  |  |  |  |  |  |  |  |  |  |  |  |  |  |  |  |  |  |
